# The complete chloroplast genome sequence of *Ficus pandurata* Hance var. *angustifolia* W.C. Cheng (Moraceae)

**DOI:** 10.1080/23802359.2022.2110005

**Published:** 2022-08-22

**Authors:** Xiaoqing Zhang, Fei Xu, Ling Guo

**Affiliations:** a College of Ecology, Lishui University, Lishui, China; b Zhejiang Provincial Key Platform of Scientific and Technological Innovation of Traditional Chinese Medicine, Lishui, China

**Keywords:** *Ficus pandurata* var. *angustifolia*, *Ficus*, chloroplast genome, phylogeny

## Abstract

*Ficus pandurata* var. *angustifolia* is an edible plant popular throughout China where it has a long history of use in traditional She medicine. Using Illumina sequencing, we assembled and annotated the complete chloroplast (cp) genome of *F. pandurata* var. *angustifolia* which is 160,526 bp and encodes 130 genes, comprised of 85 protein-coding genes, 37 transfer RNA (tRNA) genes, and eight ribosomal RNA (rRNA) genes. Phylogenetic analysis resolved *F. pandurata* var. *angustifolia* as sister to *F. deltoidea* which together formed a clade with *F. heteromorpha*. This complete cp genome sequences is a valuable resource for future studies of evolution and species delimitation in genus *Ficus* as well as variety breeding and conservation for this species.

*Ficus pandurata* Hance var. *angustifolia* W.C. Cheng 1934 is a shrub known as Xiaoxianggou in the traditional She medicine of eastern and southeastern China (Nie et al. 2016) where it is used to treat infantile malnutrition, indigestion, diarrhea, hernia, gouty arthritis, arthralgias, and other diseases (Ying et al. 2012; Wang et al. 2015; Fan et al. 2016). Many *Ficus* species are used for food and medicine and the sweet fragrance dried *F. pandurata* var. *angustifolia* material lends itself to culinary use. Therefore, *F. pandurata* var. angustifolia is widely cultivated in Zhejiang Province and has received New Resource Food Certification by the local government.

However, there are virtually no genomic resources for the study and conservation of this important species. Here, we describe the first sequenced and annotated chloroplast (cp) genome for *F. pandurata* var. *angustifolia*.

Fresh leaves of *F. pandurata* var. *angustifolia* were collected from Lishui University (28°27′N, 119°54′E) in August 2021. The specimen was deposited in Shanghai Chenshan Botanical Garden (Shanghai, China) (Binjie Ge, gebinjie@csnbgsh.cn) under accession number CSH0192340. This study was approved by the Ethics Committee of Lishui University. Total genomic DNA was extracted using the E.Z.N.A.^®^ plant DNA kit (Omega Bio-tek, Inc., Norcross, GA), the quality and integrity of extracted DNA were assessed by agarose gel electrophoresis and NanoDrop 2000 (Thermo, Inc., Waltham, MA) spectrophotometry. A DNA library with a 300–500 bp insert was constructed using the Truseq™ DNA sample Prep Kit (Illumina, Inc., San Diego, CA). Next-generation sequencing was conducted by Shanghai Origingene Bio-pharm Technology Co. Ltd. (Shanghai, China) with Illumina NovaSeq6000 platform (Illumina, Inc., San Diego, CA). About 22.65 G raw data were obtained after sequencing and filtered using FastQC (version 0.11.4) and Cutadapt (version 1.16) (Brown et al. 2017). We used the software Fast-plast (version 1.2.8) (https://github.com/mrmckain) and Geseq to assemble and annotate the cp genome (Tillich et al. 2017).

The cp genome of *F. pandurata* var. *angustifolia* was 160,526 bp in length, containing a large single-copy region (LSC) of 88,660 bp, a small single-copy region (SSC) of 20,102 bp, and two inverted repeat regions (IRs) of 25,882 bp. The overall GC content is 35.89%, and the values of the LSC, SSC, and IRs regions are 33.52%, 28.97%, and 42.62%, respectively. The genome contains 130 genes including 85 protein-coding genes, 37 transfer RNA (tRNA) genes, and eight ribosomal RNA (rRNA) genes. These values were similar to the cp genomes of other species from genus *Ficus* (Chen et al. 2020; Wang and Cui 2020; Xu et al. 2021).

In order to determine the phylogenetic position of *F. pandurata* var. *angustifolia*, 38 species with five outgroup taxa (*Broussonetia kurzii*, *Trophis scandens*, *Morus notabilis*, *Morus cathayana*, and *Antiaris toxicaria*) were used for phylogenetic analysis. Genome sequences were aligned by using MAFFT (version 7.158b) (Katoh and Standley 2013), and a maximum-likelihood phylogenetic tree was constructed by using RAxML (version 8.2.12) with 1000 bootstrap replicates (Stamatakis 2014). The molecular phylogeny revealed that the systematic position of *F. pandurata* var. *angustifolia* as sister to *F. deltoidea* which together formed a clade with *F. heteromorpha* (Figure 1). In a previous study using cp intergenic spacer data, these two species came out in a monophyletic assemblage of other species of subgenus *Ficus*, section *Ficus*, subsection Frutescentiae (Li et al. 2012). However, its autonym variety *Ficus pandurata* is found in a well-supported sister relationship to *Ficus formosana* (Figure 1), which was consistent with a recent report (Huang et al. 2022). In concordance with morphological differences between *F. pandurata* var. *angustifolia* and its autonym variety, this complete cp genome sequence of *F. pandurata* var. *angustifolia* provides insight into the genetic underpinnings of subspecific diversification in subsection Frutescentiae. This study provides the basis for future phylogenomic studies of *F. pandurata* var. *angustifolia* and the phylogenetic relationships within genus *Ficus*.

**Figure 1.**
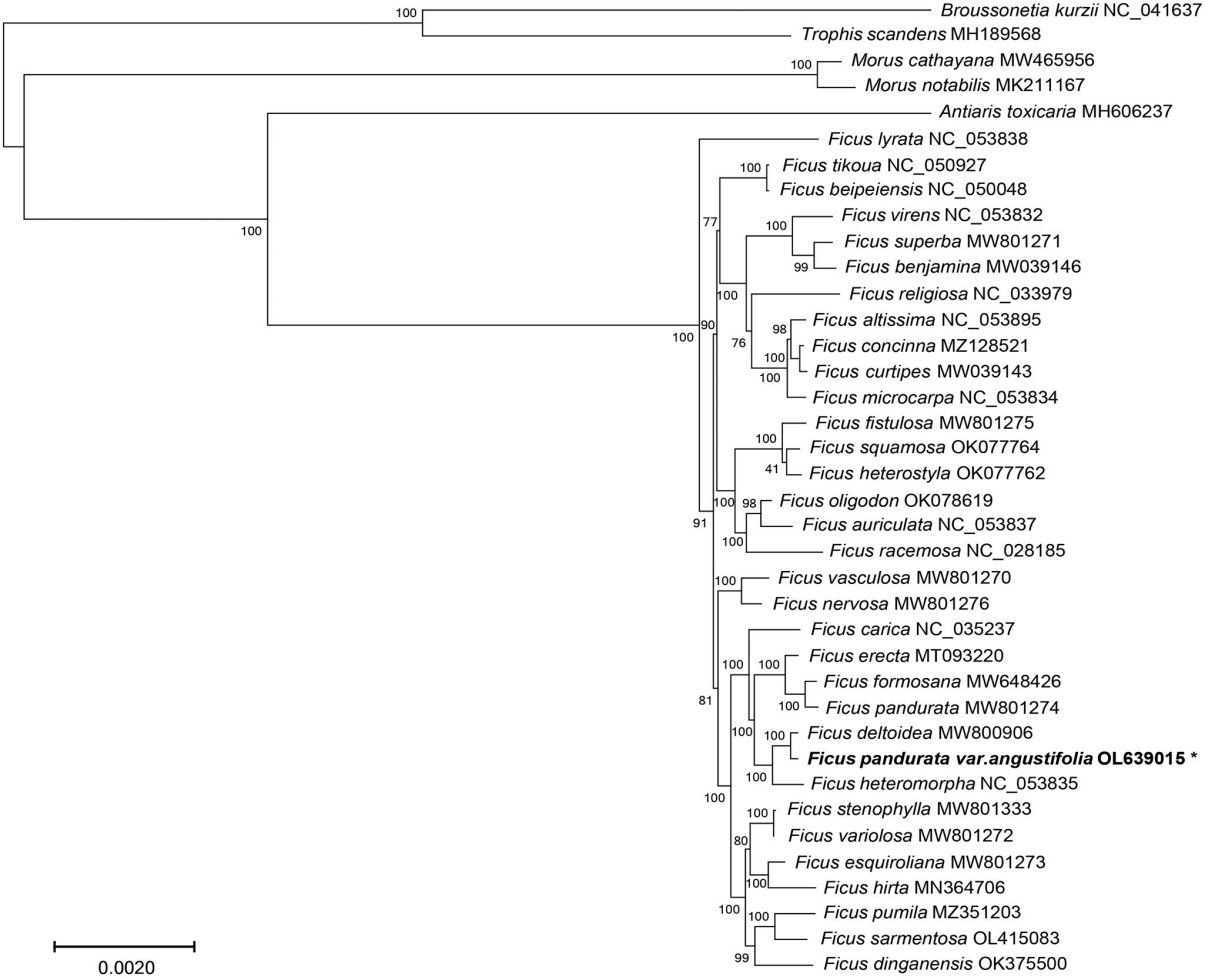
The phylogenetic tree from a maximum-likelihood analysis of complete chloroplast genome sequences from *F. pandurata* var. *angustifolia* and related species. Bootstrap support values are indicated at each node (*N* = 1000). Scale bar indicates phylogenetic distance in substitutions per site.

## Author contributions

Xiaoqing Zhang: conceptualization, methodology, writing original draft, project administration, and writing review and editing; Fei Xu: validation, resources preparing, and visualization; Ling Guo: investigation and formal analysis. All authors agree to be accountable for all aspects of the work and have approved this version to be published.

## Data Availability

The genome sequence data generated in this study are openly available in GenBank of NCBI (https://www.ncbi.nlm.nih.gov/) under the accession no. OL639015. The associated BioProject, BioSample, and SRA numbers are PRJNA783434, SAMN23429730, and SRR17035630, respectively.

## References

[CIT0001] Brown J, Pirrung M, McCue LA. 2017. FQC Dashboard: integrates FastQC results into a web-based, interactive, and extensible FASTQ quality control tool. Bioinformatics. 33(19):3137–3139. 28605449 10.1093/bioinformatics/btx373 PMC5870778

[CIT0002] Chen H, Liu C, Liu Q, Song Y, Tang L. 2020. The plastid genome of a large hemiepiphytic plants *Ficus altissima* (Moraceae). Mitochondrial DNA B Resour. 5(3):2493–2494. 33457840 10.1080/23802359.2020.1779627 PMC7781969

[CIT0003] Fan L, Yang YC, Yu HL, Chen ZJ. 2016. Research progress in twelve She medicine. China Pharmacist. 19(7):1374–1377.

[CIT0004] Huang YY, Li J, Yang ZR, An WL, Xie CZ, Liu SS, Zheng XS. 2022. Comprehensive analysis of complete chloroplast genome and phylogenetic aspects of ten *Ficus* species. BMC Plant Biol. 22(1):253. 35606691 10.1186/s12870-022-03643-4 PMC9125854

[CIT0005] Katoh K, Standley DM. 2013. MAFFT Multiple Sequence Alignment Software Version 7: improvements in performance and usability. Mol Biol Evol. 30(4):772–780. 23329690 10.1093/molbev/mst010 PMC3603318

[CIT0006] Li HQ, Wang S, Chen JY, Gui P. 2012. Molecular phylogeny of *Ficus* section *Ficus* in China based on four DNA regions. J Syst Evol. 50(5):422–432.

[CIT0007] Nie WC, Zhang XQ, Yan H, Li S, Zhu WG, Fan FY, Zhu JH. 2016. Xiaoxianggou attenuates atherosclerotic plaque formation in endogenous high Ang II ApoE/mice via the inhibition of miR-203 on the expression of Ets-2 in endothelial cells. Biomed Pharmacother. 82:173–179. 27470353 10.1016/j.biopha.2016.04.065

[CIT0008] Stamatakis A. 2014. RAxML version 8: a tool for phylogenetic analysis and post-analysis of large phylogenies. Bioinformatics. 30(9):1312–1313. 24451623 10.1093/bioinformatics/btu033 PMC3998144

[CIT0009] Tillich M, Lehwark P, Pellizzer T, Ulbricht-Jones ES, Fischer A, Bock R, Greiner S. 2017. GeSeq-versatile and accurate annotation of organelle genomes. Nucleic Acids Res. 45(W1):W6–W11. 28486635 10.1093/nar/gkx391 PMC5570176

[CIT0010] Wang Y, Cui Y. 2020. The complete plastid genome of *Ficus erecta* (Moraceae). Mitochondrial DNA B Resour. 5(3):3335–3336. 33458158 10.1080/23802359.2020.1768914 PMC7783041

[CIT0011] Wang WY, Mao JH, Yu HL, Yu L, Chen ZJ. 2015. Study on the quality standard of traditional She medicine Xiaoxianggou. Chin Arch Tradit Chin Med. 33(8):1979–1981.

[CIT0012] Xu SQ, Guo S, Fan DD, Wang JH. 2021. The complete chloroplast genome sequence of *Ficus formosana* Maxim (Moraceae) from Guangzhou, China. Mitochondrial DNA B Resour. 6(7):1895–1896. 34151011 10.1080/23802359.2021.1934170 PMC8189076

[CIT0013] Ying YY, Wang XZ, He GQ. 2012. Analysis of the nutritional components and content determination of total flavonoids in *Ficus pandurata* Hance var. angustifolia Cheng. Sci Technol Food Ind. 33(14):90–92, 99.

